# The LDL-HDL Profile Determines the Risk of Atherosclerosis: A Mathematical Model

**DOI:** 10.1371/journal.pone.0090497

**Published:** 2014-03-12

**Authors:** Wenrui Hao, Avner Friedman

**Affiliations:** 1 Mathematical Biosciences Institute, The Ohio State University, Columbus, Ohio, United States of America; 2 Mathematical Biosciences Institute & Department of Mathematics, The Ohio State University, Columbus, Ohio, United States of America; Temple University School of Medicine, United States of America

## Abstract

Atherosclerosis, the leading death in the United State, is a disease in which a plaque builds up inside the arteries. As the plaque continues to grow, the shear force of the blood flow through the decreasing cross section of the lumen increases. This force may eventually cause rupture of the plaque, resulting in the formation of thrombus, and possibly heart attack. It has long been recognized that the formation of a plaque relates to the cholesterol concentration in the blood. For example, individuals with LDL above 190 mg/dL and HDL below 40 mg/dL are at high risk, while individuals with LDL below 100 mg/dL and HDL above 50 mg/dL are at no risk. In this paper, we developed a mathematical model of the formation of a plaque, which includes the following key variables: LDL and HDL, free radicals and oxidized LDL, MMP and TIMP, cytockines: MCP-1, IFN-*γ*, IL-12 and PDGF, and cells: macrophages, foam cells, T cells and smooth muscle cells. The model is given by a system of partial differential equations with in evolving plaque. Simulations of the model show how the combination of the concentrations of LDL and HDL in the blood determine whether a plaque will grow or disappear. More precisely, we create a map, showing the risk of plaque development for any pair of values (LDL,HDL).

## Introduction

Atherosclerosis, hardening of the arteries, is the leading cause of death in the United States, and worldwide. The disease triggers heart attack or stroke, with total annual death of 900,000 in the United States [1] and 13 million worldwide [2].

Atherosclerosis is a disease in which a plaque builds up inside the arteries. A plaque contains low density lipoprotein (LDL), macrophages, smooth muscle cells (SMCs), platelets, and debris. The plaque constricts the lumen of the blood vessel thereby increasing the shear force of blood flow [3], [4]. As the plaque continues to grow, the increased shear force may cause rupture of the plaque, possibly resulting in the formation of thrombus (blood clot) [3], [5], ischemic stroke, and heart attack [3]–[5].

**Figure 1:**
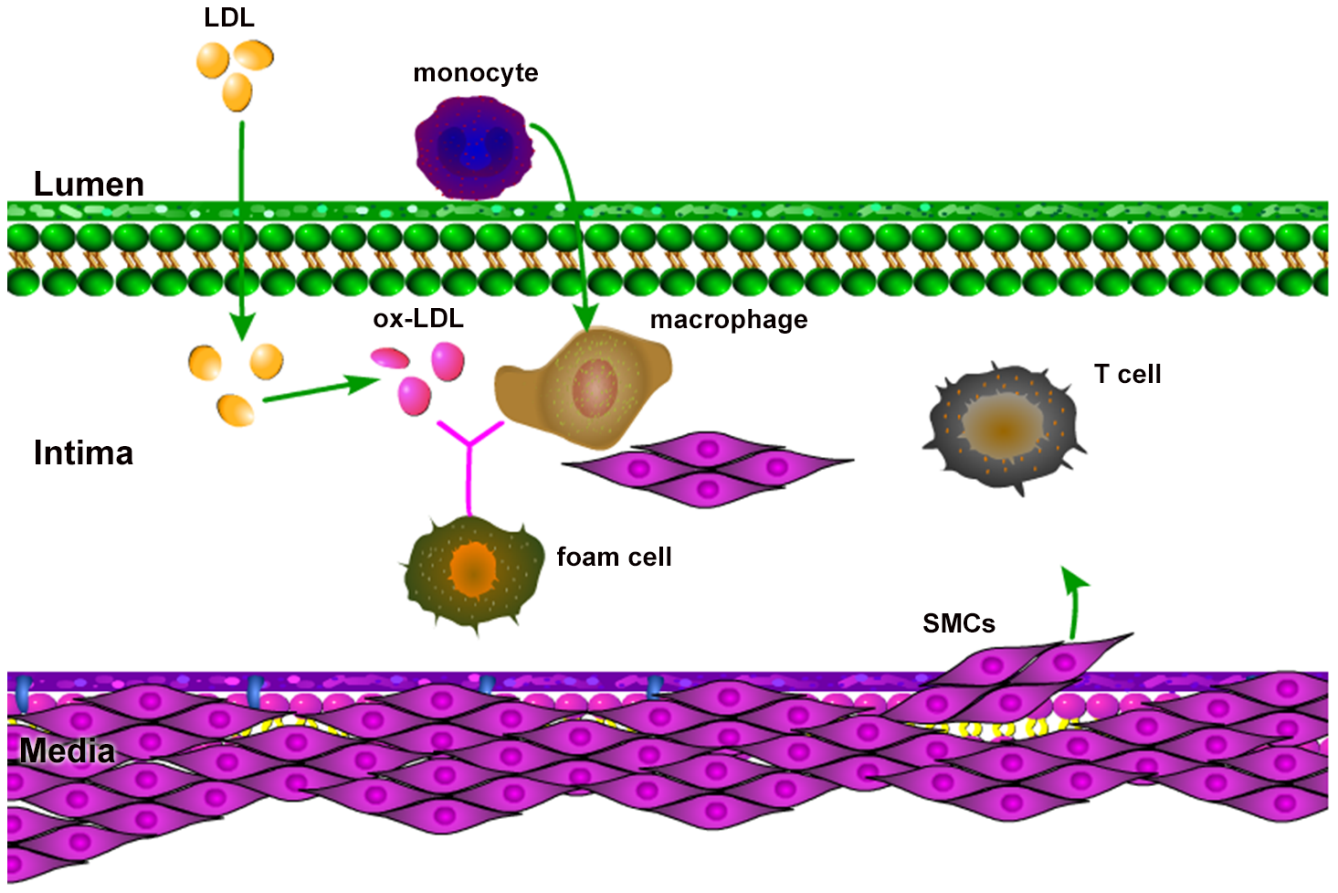
Atherosclerosis schematics: the presence of ox-LDL in the intima causes monocytes to migrate from the lumen into the intima. Monocytes differentiate into macrophages which endocytose ox-LDL and become foam cells. SMCs are attracted from the media into intima by chemotaxis and haptotaxis. Cytokines released by macrophages, foam cells and SMCs activate T cells. T cells enhance activation of macrophages. HDL helps prevent atherosclerosis.

The process of plaque development begins with a lesion in the endothelial layer, allowing LDL, to move from the blood into the intima and becoming oxidized LDL (ox-LDL) by free radicals (FRs). FRs are oxidative agents continuously released by bio-chemical reactions within the body, including the intima [6]–[8]. Endothelial cells, sensing the presence of ox-LDL, secrete monocyte chemoattractant protein (MPC-1) [9], [10], which triggers recruitment of monocytes into the intima [11]. After entering the intima, monocytes differentiate into macrophages, which have an affinity for the ox-LDL [12]–[14]. The ingestion of large amounts of ox-LDL transforms the fatty macrophages into foam cells [12], [15]. Foam cells secrete chemokines which attract more macrophages [10], [12], [13]. SMCs from the media move into the intima by chemotactic forces due to MCP-1 [9], [10], and platelet-derived growth factor (PDGF) [10], [16], as well as by haptotaxis by the extracellular matrix (ECM). PDGF is secreted by macrophages, foam cells and SMCs [16], [17]. ECM is remodeled by matrix metalloproteinase (MMP) produced by a variety of cell types including SMCs [18], and is inhibited by tissue inhibitor of metalloproteinase (TIMP) produced by macrophages and SMCs [19]. Interleukin IL-12, secreted by macrophages and foam cells [10], [12], [20], contribute to the growth of a plaque by activating T cells [9], [20], [21]. Indeed, the activated T cells secrete interferon IFN-*γ*, which in turn activates macrophage in the intima [13], [21], [22]. At the same time that LDL enters the intima, high density lipoprotein (HDL) also enters into the intima, and becomes oxidized by free radicals [7], [8]. However, oxidized HDL (ox-HDL) is not ingested by macrophages. HDL helps prevent atherosclerosis by removing cholesterol from foam cells, and by the limiting inflammatory processes that underline atherosclerosis [23]. Furthermore, HDL takes up free radicals that are otherwise available to LDL. Some of the key players in the atherosclerosis process are shown in Fig. 1.

**Figure 2:**
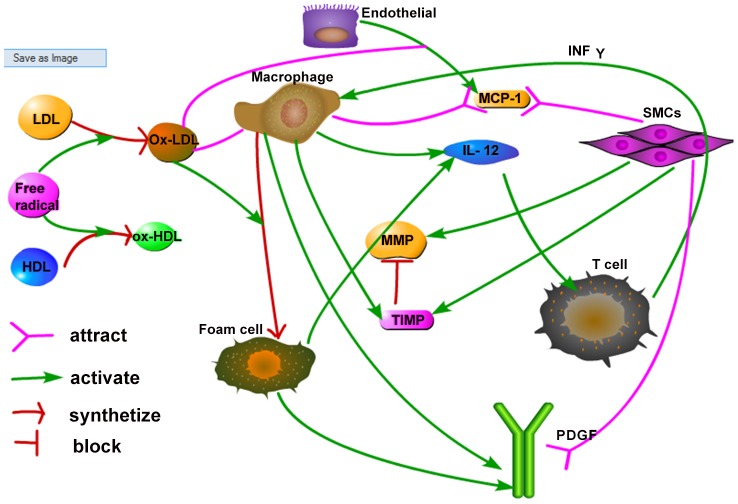
Schematic network of atherosclerosis. LDL and HDL are oxidized by free radicals, and become ox-LDL and ox-HDL respectively. Ox-LDL recruits macrophages to intima. By ingesting ox-LDL, macrophages are transformed to foam cells. SMCs are attracted into the intima by MCP-1 (secreted by endothelial cells) and PDGF (secreted by macrophages and foam cells). Macrophages, foam cells and SMCs secrete IL-12, which activates T cells. IFN-*γ* secreted by T cells enhance the activity of macrophages which contributes the plaque built-up.

It has long been recognized that the cholesterol concentrations in the blood are indicators of the probability that a plaque will develop: higher LDL and lower HDL concentrations indicate a higher probability of plaque development. Public health guidelines in the U.S. specify what levels of LDL are low risk and what levels are high risk; they also specify what levels of HDL are poor and what levels are near ideal [24], [25]. However, what is more relevant is to specify the risk associated with combined levels of LDL and HDL, and this is what the present paper addresses. A schematic of the network of atherosclerosis is given in Fig. 2. In this paper, we developed a mathematical model of plaque formation by a system of partial differential equations based on Fig. 2. The aim of the model is to determine the risk of plaque formation for combined levels of LDL and HDL. In particular, we created a “risk-map” for plaque development in the LDL-HDL coordinate plane, where the first quadrant of the plane was divided into regions of high risk, low risk and no risk. Anti-cholesterol drugs are aimed at lowering high levels of LDL, but some drugs are known to also increase the level of HDL [24]. Hence such a risk-map may be important when evaluating the extend to which an anti-cholesterol drug can reduce the risk of atherosclerosis for particular individuals.

## Materials and Methods

### Mathematical model

**Figure 3:**
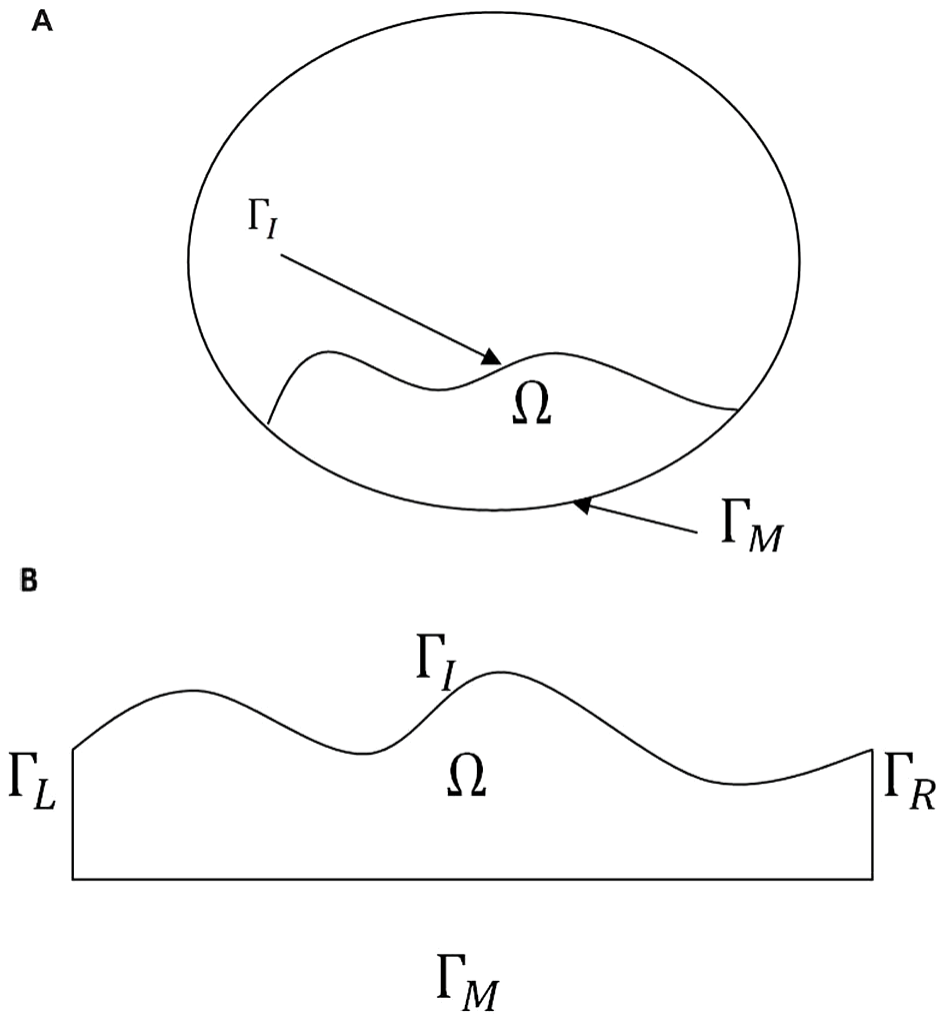
Two 2D cross sections of a plaque. Γ*_M_* is the boundary of the intima in contact with the media, and Γ*_I_* is the boundary of the intima in contact with the lumen. In (B) Γ*_L_* and Γ*_R_* are parts of the intima.

In this paper, we present a mathematical model based on the network shown in Fig. 2. The model includes the variables listed in Table 1. We assume that all cells are moving with a common velocity **u**; the velocity is the result of movement of macrophages, T cells and SMCs into the intima. We also assume that all species are diffusing with appropriate diffusion coefficients. The equation for each species of cells *X* has a form
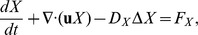
where the expression on the left-hand side includes advection and diffusion, and *F_X_* accounts for various growth factors, bio-chemical reactions, chemotaxis and haptotaxis. The equation for the chemical species are the same but without the advection term. Fig. 3 shows a 2D cross section of a blood vessel with plaque Ω, and a planar cross section of a plaque in the direction along a blood vessel.

**Table 1:** The variables of the model: concentrations and densities are in units of *g/cm* ^3^.

*L*:	concentration of LDL	*H*:	concentration of HDL
*L_ox_*:	concentration of ox-LDL	*r*:	concentration of free radicals
*P*:	concentration of MCP-1	*I_γ_*:	concentration of IFN-*γ*
*I*_12_:	concentration of IL-12	*G*:	concentration of PDGF
*Q*:	concentration of MMPs	*Q_r_*:	concentration of TIMP
*M*:	density of macrophages	*T*:	density of T cells
*S*:	density of SMCs	*ρ*:	density of ECM
*F*:	density of foam cell	*σ*:	pressure (in *g cm* ^2^/*day*)
**u**:	fluid velocity (in *cm/day*)		

#### Equations for lipoproteins [LDL (*L*), HDL (*H*), ox-LDL (*L_ox_*)] and free radical (*r*)

The distribution of LDL, HDL, ox-LDL and free radicals in the intima are described using reaction-diffusion equations [7],
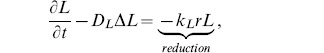
(1)

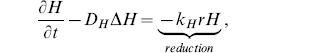
(2)

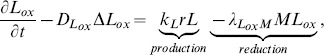
(3)

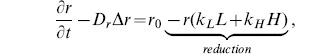
(4)where *k_L_* and *k_H_* are reaction rates of oxidization, and 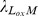 is the reduction rate of ox-LDL due to ingestion by macrophages. Eqs. (1) and (2) model the evolution of LDL and HDL concentrations. It is assumed that LDL and HDL are lost by reaction of oxidation with free radicals. Equation (3) models the production of ox-LDL due to LDL oxidation by reaction with the radicals (first term on right-hand side) and a reduction of ox-LDL through ingestion by macrophages (second term on right-hand side). Equation (4) models the evolution of free radicals concentration with baseline growth *r*_0_.

#### Equation for macrophages (*M*)

The evolution of macrophage density is modeled by
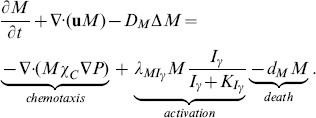
(5)Here the first term on right-hand side accounts for recruitment of macrophages by MCP-1 [9], and the second term accounts for the activation of macrophages by IFN-*γ* [13], [21], [22].

#### Equation for MCP-1 (*P*)

The MCP-1 equation is given by
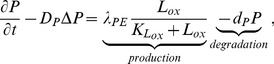
(6)where the first term on the right-hand side is the production of MCP-1 by endothelial cells, whose density is assumed to be constant, under the influence of ox-LDL [9].

#### Equation for T cells (*T*)

The density of T cells, which are primarily CD4^+^ T cells [20], satisfies the equation
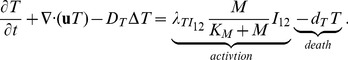
(7)In this equation, we assume that T cells are activated by IL-12 in conjunction with MHC-II (major histocompatibility complex, class II). Actually, T cells are also activated by IL-1 and IL-6 produced by macrophages and SMCs [10], [12], [13]. However, because of lack of experimental data, we do not include the IL-1 and IL-6 explicitly but instead consider their effect implicitly in estimating the parameter 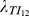. For simplicity, we include the anti-inflammatory effect of IL-10 produced by macrophages only implicitly, by the factor 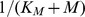.

#### Equation for IFN-*γ* (*I_γ_*)

The dynamics of IFN-*γ* concentration is modeled by
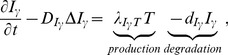
(8)where the first term on right-hand side represents production of *I_γ_* by T cells [21].

#### Equation for SMCs (*S*)

The equation of the SMCs density is given by
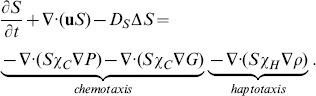
(9)The first two terms on right-hand side account for chemotaxis by MCP-1 [9], [10], and PDGF [10], [16], and the last term accounts for haptotaxis by ECM.

#### Equation for IL-12 (*I*_12_)

The concentration of IL-12 is modeled by
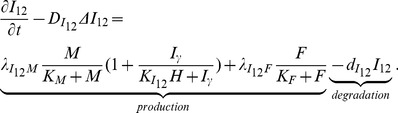
(10)The first term of right-hand side is the production of *I*_12_ by macrophages enhanced by *I_γ_* and resisted by HDL [23]. The production of *I*_12_ by macrophages is resisted by *I*_10_ (which, for simplicity, is accounted by the factor 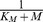) [10], [12]. The second term represents the production of *I*_12_ by foam cells [20].

#### Equations for PDGF (*G*), MMP (*Q*) and TIMP (*Q_r_*)

We have the following sets of reaction diffusion equations for the chemokines (*G*, *Q* and *Q_r_*):
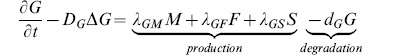
(11)

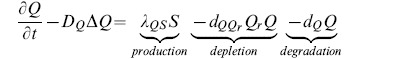
(12)

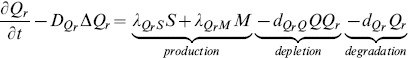
(13)In equation (11), PDGF is produced by macrophages, foam cells, and SMCs [16], [17]. In equation (12), MMP is secreted by SMCs [18] (first term on right-hand side), and is lost by binding with TIMP (second term). In equation (13), TIMP is produced by SMCs and macrophages [19].

#### Equation for foam cells (*F*)

Macrophages that have ingested a large amount of ox-LDL become foam cells [7], [12], [15], so we have
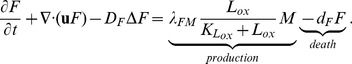
(14)


#### Equations for ECM (*ρ*) and pressure (*σ*)

We assume that the intima has the constituency of a porous medium. Then, by Darcy's law, the velocity **u** of the cells is given by
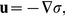
(15)where *σ* is the pressure. We also assume that the total density of all the cells plus the concentration of *ρ* is constant. This constant should be smaller than the average density of a plaque, 1.22±0.03 g/*cm* ^3^ [26], because plaques contain some debris, which are not included in our model. We take the constant to be 1 g/*cm* ^3^, i.e.,
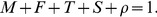
(16)We assume that all cells are approximately of the same volume and surface area, so that the diffusion coefficients of the all cells have the same coefficient, *D*. By adding Eqs. (5), (7), (9) and (14), we get
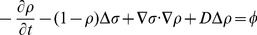
(17)where
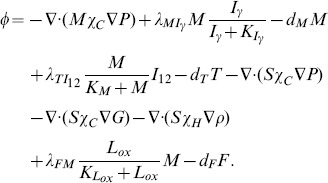
(18)
Eq. (17) gives a relation between *ρ* and *σ*. We next derive an equation for *ρ*. The ECM is degraded by MMP [27], and is remodeled by macrophages and SMCs [18], [27]. For simplicity, we take the remodeling rate to be a constant, *λ_ρ_*, as in [28]. Then the equation of the density of ECM is given by
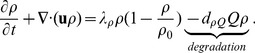
(19)Since 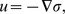 this equation can be written in the form
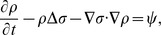
where
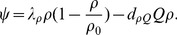
(20)


Adding this equation and (17), we get our final equation for *σ*:
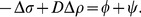
(21)The equation for 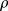 can then be written as
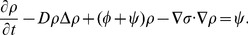
(22)


### Boundary conditions

For simplicity, we consider only 2-dimensional plaques as in Fig. 3. Then the boundary of the plaque consists of i) Γ*_M_*, in contact with media; ii) a free boundary Γ*_I_*, inside the lumen, and iii) two more vertical boundaries Γ*_L_* and Γ*_R_* of the intima in the case of Fig. 3(B).

#### Boundary conditions on Γ*_I_*

We assume flux boundary conditions of the form
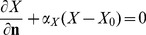
(23)for *X* = *L*, *H*, *M*, *T*, and non-flux boundary conditions for all other variables,
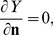
(24)where 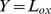, *r*, *P*, *I_γ_*, *S*, *I*, *I*_12_, *G*, *Q*, *Q_r_*, *F*. The boundary values for *ρ* are determined by Eq. (16). The coefficient 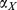 is a constant except for *M*, and 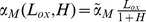, since ox-LDL attracts monocytes [11], while HDL limits the inflammation process[23]. Note that *L*_0_ and *H*_0_ are the LDL and HDL concentrations in the blood, so we shall be interested to see how these concentrations determine whether a small plaque will grow or shrink.

As in [29]–[31], we assume that the free boundary Γ*_I_* is held together by cell-to-cell adhesion forces so that
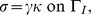
where *κ* is the mean curvature of the surface Γ*_I_*. (If Γ*_I_* is circular, then *κ* is the reciprocal of the radius) Furthermore, the continuity condition 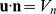, where **n** is the outward normal and *V_n_* is the velocity of the free boundary Γ*_I_* in the direction **n**, yields the relation
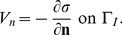
(25)


#### Boundary conditions on Γ*_M_*

We assume non-flux boundary conditions for all variables except *ρ* and *S* on Γ*_M_*: For *S*, we have
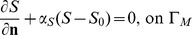
where *S*_0_ is SMCs density in the media, and for simplicity we take 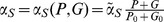 since MCP-1 and PDGF attract SMCs from the media. As in the case of Γ*_I_*, the boundary values of *ρ* are determined by Eq. (16).

#### Boundary conditions on Γ*_L_* and Γ*_R_*

We assume the periodic boundary conditions on Γ*_L_* and Γ*_R_*.

### Parameter estimation

Table 2 lists the range of molecular weights of proteins and Table 3 lists their range of concentration. In the second columns in Tables 2 and 3, we indicate the (intermediate) values used in the simulations. The Tables 2 and 3 are used to estimate some of the model parameters. A summary of all the model parameters is given in Tables 4 and 5.

**Table 2:** Molecular weights.

Protein	Weight (kda)	Explanation
LDL	549	Over 95% of the LDL protein mass is apolipoprotein
		B-100 (apo B-100, 549 kDa (1000 g/mol)) [63].
HDL	105	The range of weight of HDL is 105–130 [64].
Free radical	0.51 kda	Free radicals include DPPH (0.39 kda),
		ABTS (0.51 kda) and superoxide anion (0.81 kda) [65].
IFN-*γ*	17	IFN-*γ* is described as a 17 kDa peptide [66].
PDGF	35	There are two PDGF polypeptides:
		PDGF-I with a molecular weight of about 35 kda, and
		PDGF-II with a molecular weight of about 32 kda [67].
MCP-1	8.9	[68]
IL-12	70	[69]
MMP	52	MMP-1 has two major species of molecular
		mass, 57 kDa and 52 kDa [48].
TIMP	25	The molecular weights of TIMP-1, TIMP-2 and TIMP-3
		are 28.5 kDa 21 kDa and 27 kDa respectively [48].

**Table 3:** Concentrations of proteins and cells.

Proteins & cells	Concentration (*gcm* ^−3^)	Explanation
LDL	7×10^−4^–1.9×10^−3^	Range is 70–190 mg/dl [25], [70].
HDL	4×10^−4^–6×10^−4^	Range is 40–60 mg/dl [25], [70].
IFN-*γ*	10^−9^	Range is of 0.1–10.0 ng/mL [10].
PDGF	1.5×10^−8^	Range in normal humans blood
		17.5±3.1 ng/mL [72].
MCP-1	3×10^−10^	300 pg/ml [73]
IL-12	5×10^−10^	Range 200–800 pg/ml [20].
MMP	3×10^−8^	Range in plasma is 10∼60 ng/ml [74].
TIMP	3×10^−8^	Range in plasma is 10∼60 ng/ml [74]
SMC	6×10^−3^	Range 7,500,000–10,000,000 cells per ml [38].
Monocyte	5×10^−5^	Range from 20,000 to 100,000 cells per ml [75].
T cell	1×10^−3^	Range of CD4^+^ T cells in healthy normal adult
		of 500,000 to 1,500,000 cells per ml [49].

**Table 4:** Parameters' description and value.

Parameter	Description	Value
*k_L_*	reaction rate of LDL + Radical→ox-LDL	2.35×10^−4^ *g* ^−1^ *cm* ^3^ day^−1^ [7], [34], [35]
*k_H_*	reaction rate of HDL + Radical→ox-HDL	5.29×10^−6^ *g* ^−1^ *cm* ^3^ day^−1^ [7], [34]
*D_L_*	diffusion coefficient of LDL	29.89 *cm* ^2^ day^−1^ [33], [34], [37] & estimated
*D_H_*	diffusion coefficient of HDL	3.93 *cm* ^2^ day^−1^ [33], [34], [37] & estimated
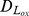	diffusion coefficient of oxidized LDL	29.89 *cm* ^2^ day^−1^ [33], [34], [37] & estimated
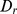	diffusion coefficient of radicals	2.05×10^−1^ *cm* ^2^ day^−1^ [33], [34], [37] & estimated
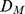	diffusion coefficient of macrophage	8.64×10^−7^ *cm* ^2^ day^−1^ [28], [36]
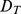	diffusion coefficient of T-cell	8.64×10^−7^ *cm* ^2^ day^−1^ [28], [36]
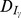	diffusion coefficient of IFN-*γ*	1.08×10^2^ *cm* ^2^ day^−1^ [76]
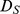	diffusion coefficient of SMCs	8.64×10^−7^ *cm* ^2^ day^−1^ [28], [36]
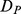	diffusion coefficient of MCP-1	17.28 *cm* ^2^ day^−1^ [44]
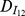	diffusion coefficient of IL-12	1.08×10^2^ *cm* ^2^ day^−1^ [76]
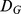	diffusion coefficient of PDGF	8.64×10^−2^ *cm* ^2^ day^−1^ [42]
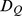	diffusion coefficient of MMP	4.32×10^−2^ *cm* ^2^ day^−1^ [38]
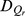	diffusion coefficient for TIMPs	4.32×10^−2^ *cm* ^2^ day^−1^ [33], [37], [38] & estimated
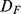	diffusion coefficient of foam cells	8.64×10^−7^ *cm* ^2^ day^−1^ [28], [36]
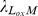	rate of ox-LDL ingestion by macrophages	10 *gcm* ^−3^ day^−1^ [7]
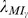	activation rate of macrophages by IFN-*γ*	0.005 day^−1^ [39] & estimated
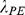	production rate of MCP-1	8.65×10^−10^ *gcm* ^−3^ day^−1^ [44] & estimated
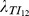	activation rate of T cells by IL-12	1×10^6^ day^−1^ [39], [40], [49], [74] & estimated
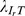	production rate of IFN-*γ* by T cells	0.066 day^−1^ [45], [77]
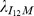	production rate of IL-12 by macrophages	3×10^−7^ *gcm* ^−3^ day^−1^ [45]
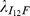	production rate of IL-12 by foam cells	1×10^−7^ *gcm* ^−3^ day^−1^ [45] & estimated
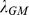	production rate of PDGF by macrophages	0.1 day^−1^ [41], [42] & estimated
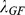	production rate of PDGF by foam cells	0.033 day^−1^ [41], [42] & estimated
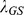	production rate of PDGF by SMCs	0.5 day^−1^ [41], [42] & estimated
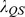	production rate of MMP by SMCs	3×10^−4^ day^−1^ [28]
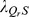	production rate of TIMP by SMCs	3×10^−5^ day^−1^ [28] & estimated
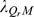	production rate of TIMP by macrophages	6×10^−5^ day^−1^ [28] & estimated
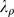	remodeling rate of ECM	0.432 day^−1^ [28]
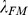	activation rate of foam cells	0.12 day^−1^ [39] & estimated
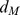	death rate of macrophage	0.015 day^−1^ [39]
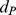	degradation rate of MCP-1	1.73 day^−1^ [44]
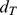	death rate of T cell	0.33 day^−1^ [39], [40]
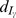	degradation rate of IFN-*γ*	0.69 day^−1^ [37]
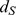	death rate of SMC	0.86 day^−1^ [7]
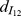	degradation rate of IL-12	1.188 day^−1^ [39], [40]
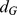	degradation rate of PDGF	3.84 day^−1^ [42]
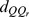	binding rate of MMP to TIMP	4.98×10^8^ *cm* ^3^ *g* ^−1^ day^−1^ [44], [48] & estimated
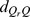	binding rate of TIMP to MMP	1.04×10^9^ *cm* ^3^ *g* ^−1^ day^−1^ [44], [48] & estimated
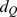	degradation rate of MMP	4.32 day^−1^ [37]
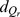	degradation rate of TIMP	21.6 day^−1^ [46] & estimated
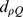	degradation rate of ECM due to MMP	2.59×10^7^ *cm* ^3^ *g* ^−1^ day^−1^ [36]
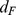	death rate of foam cell	0.03 day^−1^ [39] & estimated

**Table 5:** Parameters' description and value.

Parameter	Description	Value
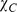	chemotactic sensitivity parameter	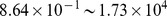 *cm* ^5^ *g* ^−1^ day^−1^ [36], [37] (10)*
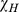	haptotaxis parameter	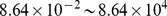 *cm* ^5^ *g* ^−1^ day^−1^ [36], [37] (10^2^)*
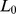	source/influx of LDL in blood	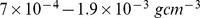 [25]
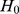	source/influx of HDL in blood	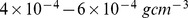 [25]
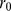	source/influx of free radical into intima	0.26 *gcm* ^−3^ day^−1^ [34]
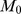	source/influx of macrophages from blood	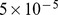 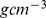 [75]
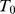	source/influx of T cells into intima	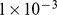 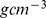 [49]
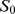	source/influx of SMCs into intima	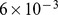 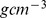 [71]
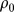	ECM density	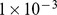 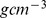 [28]
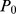	MCP-1 concentration	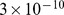 [73]
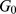	PDGF concentration	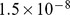 [72]
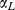	influx rate of LDL into intima	1.0 *cm* ^−1^ estimated
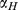	influx rate of HDL into intima	1.0 *cm* ^−1^ estimated
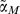	influx rate of macrophage into intima	0.2 *cm* ^−1^ estimated
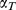	influx rate of T cells into intima	0.05 *cm* ^−1^ estimated
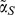	influx rate of of SMCs into intima	0.2 *cm* ^−1^ estimated
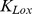	ox-LDL saturation for production of MCP-1	0.5 *gcm* ^−3^ [64] & estimated
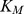	macrophages saturation for activation of T cells	2.5×10^−5^ *gcm* ^−3^ [75] & estimated
	and production of IL-12	
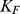	foam cells saturation for production of IL-12	2.5×10^−5^ *gcm* ^−3^ [75] & estimated
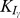	IFN-*γ* saturation for activation of macrophages	1×10^−11^ *gcm* ^−3^ [39]
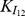	IFN-*γ* saturation for production of IL-12	7×10^−11^ *gcm* ^−3^ [45]

* Values chosen in the simulation.

#### Reaction rates

To estimate some of the parameters in the equations for proteins, we shall use the concept of “accessible surface area” [32], [33] of a protein *p*, or briefly “area,” 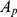, which is roughly the minimum surface area of the smooth shapes containing the protein. It was estimated in [34], that 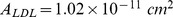, and 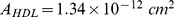, so that their ratio is
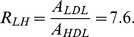
Accordingly, the corresponding reaction rates of the oxidation, *k_L_* and *k_H_*, are related by 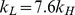. Moreover, the reaction rate of oxidation of LDL by free radicals is 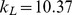 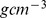 day^−1^ [7], [34], [35], so that 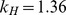 g *cm* ^−3^ day^−1^.

#### Diffusion coefficients

We assume that the diffusion coefficients of all the cells are the same, and take them to be 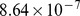 *cm* ^2^ day^−1^ [28], [36]. In order to estimate the diffusion coefficients of the various proteins, we assume that the diffusion coefficient of protein *p*, *D_p_*, is proportional to its area *A_p_*, i.e., 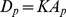, where we take *K* to be the same for all small molecules. For glucose, which is a monomeric globular protein, 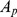 can be computed in terms of the molecular weight 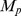, by the formula 
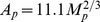
 [33], and 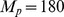 dalton [28], 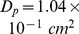 day^−1^ [37]. Hence, for glucose, *K* is determined by
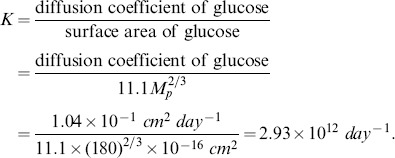
(26)We can now compute 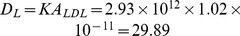 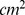 day^−1^ and 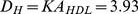 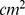 day^−1^. Free radicals are monomeric globular proteins (average weight is 500 da, Table 2). Hence
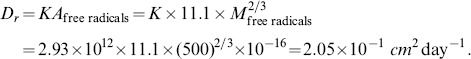
The diffusion coefficient of MMP is 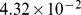 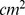 day^−1^ [38]. We assume that the diffusion coefficient of TIMP is same as that of MMP.

#### Production rates

We assume that, in Eq. (7), 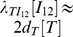, where 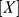 denotes the average concentration of *X*. We take 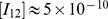 gcm^−3^, 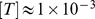 gcm^−3^ from Table 3, and 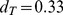 day^−1^ [39], [40]. Then 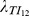 is estimated by 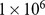 day^−1^.

PDGF is produced by SMCs, and likely also by endothelial cells and macrophages [41]. In wound healing, macrophages produce PDGF at rate of 5.76 day^−1^ [42]. Since the plaque formation is a much slower process, we take this rate 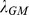 to be much smaller, i.e., 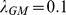 day^−1^. Since SMCs production rate of PDGF is higher than that by macrophages [41], we take 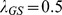 day^−1^.

The production rate of MMP by tumor cells was estimated in [28] to be 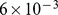 day^−1^. We assume that SMCs produce MMP at a much lower rate, namely, 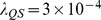 day^−1^. Since SMCs produce MMP to enable them move into the intima by haptotaxis, we assume that they produce TIMP at a lower rate than MMP, and take 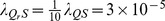 day^−1^. As macrophages produce most of the TIMP [43], we take the production rate of TIMP by macrophages to be 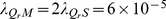 day^−1^.

We assume that the production rate of MCP-1 by endothelial cells, 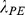, is twice that of 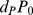, where 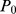 is the concentration of MCP in the blood, which is equal to 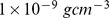 [44]. We assume that 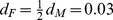 day^−1^ [39], and that, in Eq. (14), 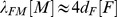 and 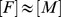, so that 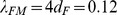 day^−1^.

By [45], 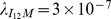 day^−1^. We assume that foam cells have lower production rates of *I*_12_ and PDGF than macrophages, and take 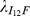 and 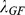 to be one third of the values of 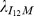 and 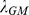, respectively, so that 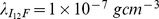 day^−1^, and 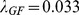 day^−1^.

#### Degradation rates

The degradation rate of MMP is 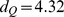 day^−1^ [37]. Since TIMP has a short half life compared to MMP [46], we take its degradation rate to be 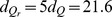 day^−1^.

In [47], the binding rate of MMP and TIMP is reported to be 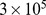 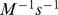, where 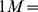
the mass per mole, and the molecular weights of MMP and TIMP are 52 kda and 25 kda, respectively [48]. Accordingly, we derive the binding rate per Molar per second (by same formula as in [44]),
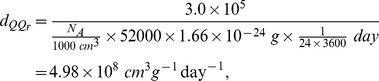
and
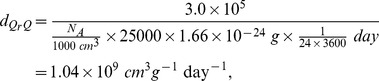
where *N_A_* is called the Avogadro number, and is the number of molecular per *dm* ^3^. 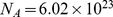, and 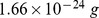 is the mass of a proton for atomic mass unit.

#### Other parameters

The range of macrophages in the blood is 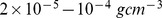 [75]; we take 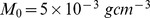. The range of T cells in the blood is 
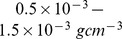
 [49]; we take 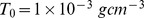. The range of SMCs is 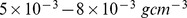 [38]; we take 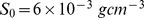. We assume that 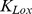 is half of 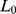 in Eq. (6), and similarly, 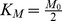 in Eq. (7), and 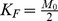 in Eq. (10). We assume that the influx of LDL and HDL into the intima is larger than the influx of macrophages and SMCs, and take 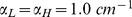, and 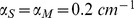. The influx of T cells is assumed to be smaller than that of macrophages, and we take 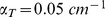.

### Numerical methods

#### Finite element implementation

In order to illustrate our numerical method, we consider the following diffusion equation with Robin boundary conditions:
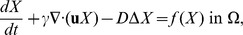


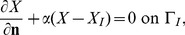
(27)

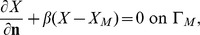
where 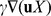 is an advection term, and either 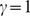 or 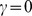 (no advection), and 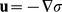. Multiplying the differential equation by an arbitrary function 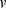, and performing integration by parts using the boundary conditions, we get
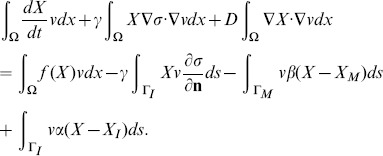
(28)


This is an equivalent formulation of the system (27), which is better suited for simulation.

Similarly, Eq. (21) for *σ* has the equivalent form:
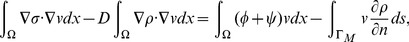
(29)for an arbitrary function 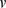.

#### Galerkin discretization

The standard Galerkin discretization method uses finite dimensional subspaces 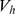 to approximate the solution *X*. Let 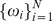 be a basis of 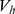, where *N* is the number of nodes within the triangulation *K*. Let 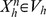 denote the numerical approximation of *X* at time 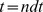, where *dt* is the time step, 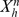 is written as a sum
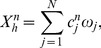
(30)for coefficient 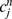 to be determined. If 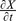 is approximated by 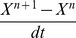, then (28) is equivalent to
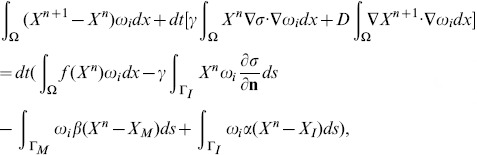
(31)or,
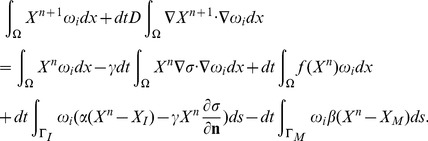
(32)


Recalling (30), we can rewrite the system (32) as a linear system of equations
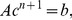
(33)where 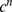 is the vector of 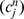, and the coefficient matrix 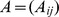 and the right-hand side 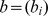 are defined by
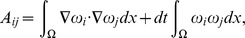
and
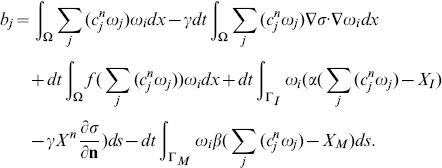
Similarly, setting 
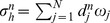
, where 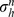 is a numerical approximation of *σ* at time *ndt*, Eq. (29) can be written as follows
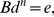
(34)where 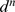 is the vector 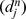, *B* is a matrix 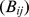, 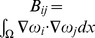, and 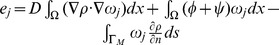.

#### Outline of the procedure

Suppose the domain Ω(*t*) has polygonal boundaries Γ*_I_*(*t*) and Γ*_O_*(*t*). Then we can cover the closure 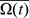 of 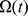 by a regular triangulation 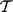 of triangles, i.e., 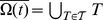 where each *T* is a closed triangle. The triangular mesh, which is a basic thing that Finite Elements requires, is generated by distmesh [50], which is a mesh generation tool implemented in MATLAB, and our algorithm is outlined in **Algorithm S1**. For the detailed implementations, such as: construct basis functions over the triangulation, assemble the stiffness matrix, etc, see references [51], [52].

## Results

**Figure 4:**
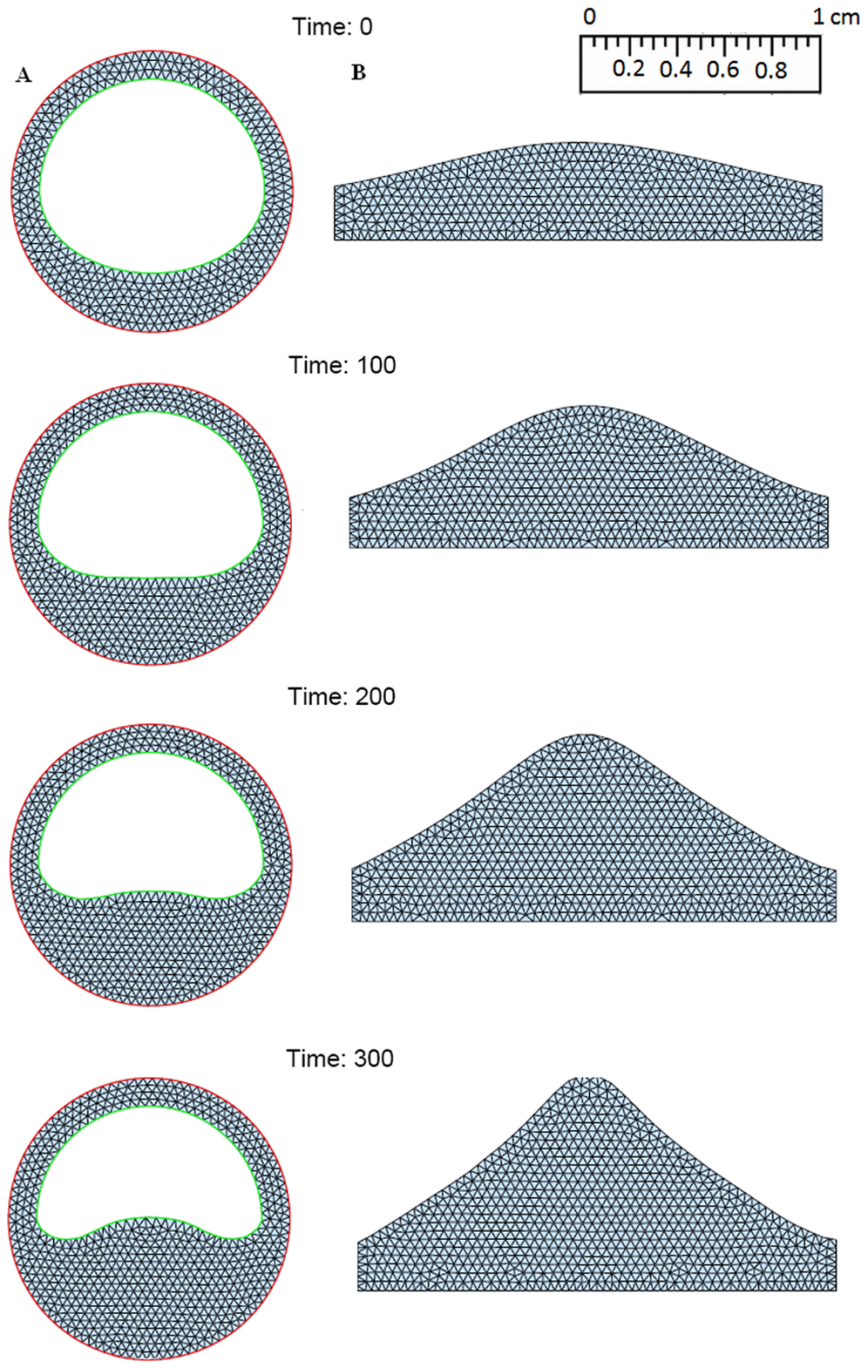
Simulations for the atherosclerosis model of 300 days after an initial plaque is formed with *H*_0_ = 40 mg/dL and *L*_0_ = 190 mg/dL. (A: Cross sections of a blood vessel, B:Cross sections along the blood vessel).

**Figure 5:**
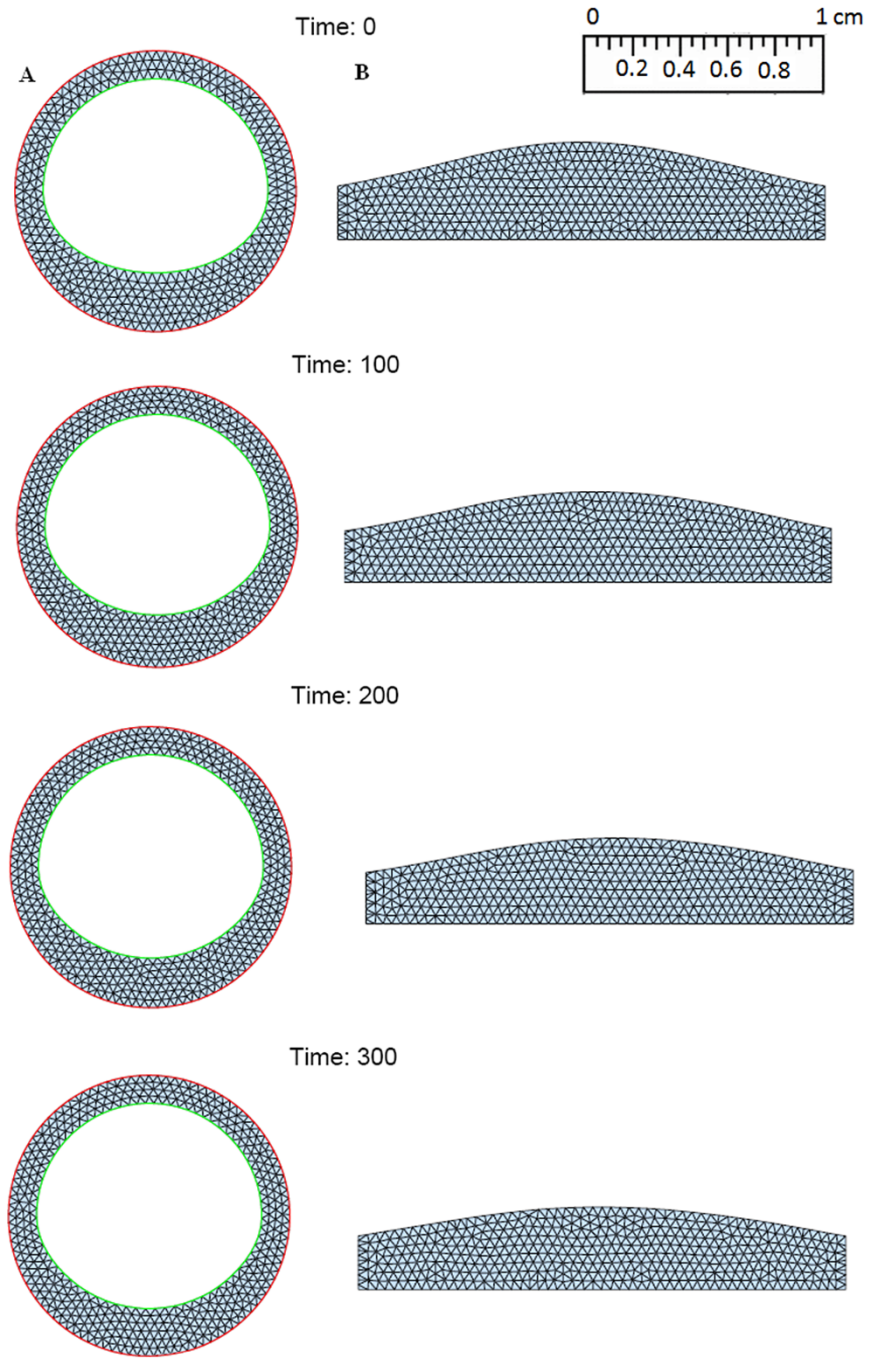
Simulations for the atherosclerosis model of 300 days after an initial plaque is formed with *H*_0_ = 50 mg/dL and *L*_0_ = 130 mg/dL. (A: Cross section of a blood vessel; B: Cross section along the blood vessel).

**Figure 6:**
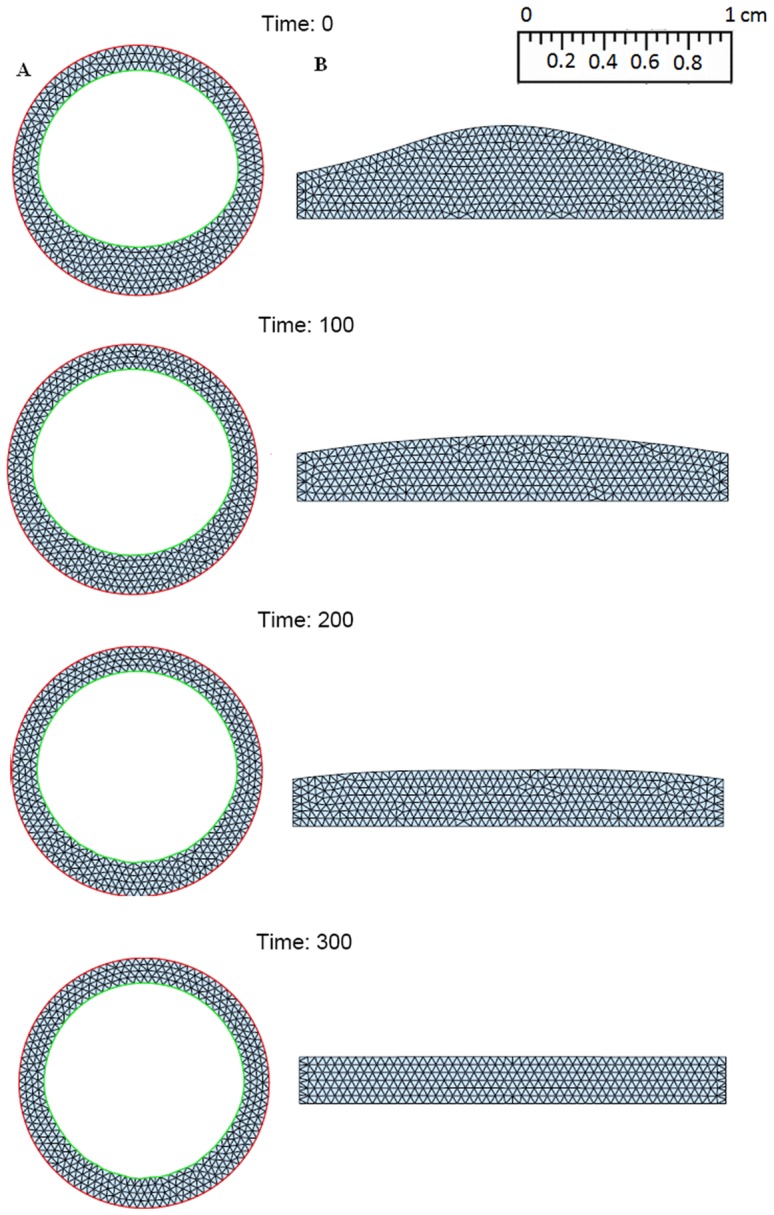
Simulations for the atherosclerosis model of 300 days after an initial plaque is formed with *H*_0_ = 60 mg/dL and *L*_0_ = 70 mg/dL. (A: Cross section of a blood vessel; B: Cross section along the blood vessel).

Numerical simulation is initialized by a small formed plaque. (see Figs. 4, 5, 6). Five combined levels of LDL and HDL (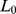 and 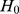) are tested for 300 days:

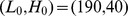
: a small plaque doubles in size at 300 days;

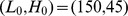
: a small plaque increases approximately 50% at 300 days;

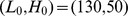
: a small plaque remains small at 300 days;

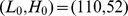
: a small plaque decreases approximately 30% at 300 days;

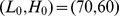
: a small plaque almost disappear at 300 days.

**Figure 7:**
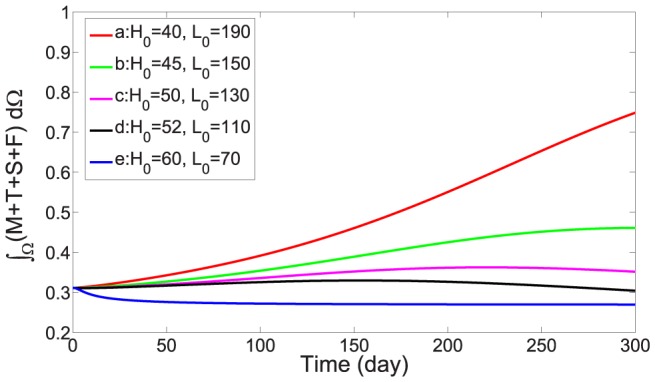
Plaque weights for different levels of LDL and HDL. The units of *H*_0_ and *L*_0_ are mg/dL.

Fig. 4 shows the growth of the plaque in case (a), Fig. 5 shows the shrinkage of the plaque in case (c), and Fig. 6 shows almost no plaque in case (e). In Fig. 7, the weight of the plaque, the summation of total cells, namely, 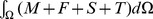, is plotted for these five scenarios of combined levels of LDL and HDL. Similarly to Fig. 7, we show in **supporting information files** how the populations of macrophages, SMCs, foam cells and T cells, as well as the concentration of ox-LDL, IFN-*γ* and IL-12, vary for different levels of LDL and HDL shown in Figs. S1, S2, S3, S4, S5, S6, S7.

**Figure 8:**
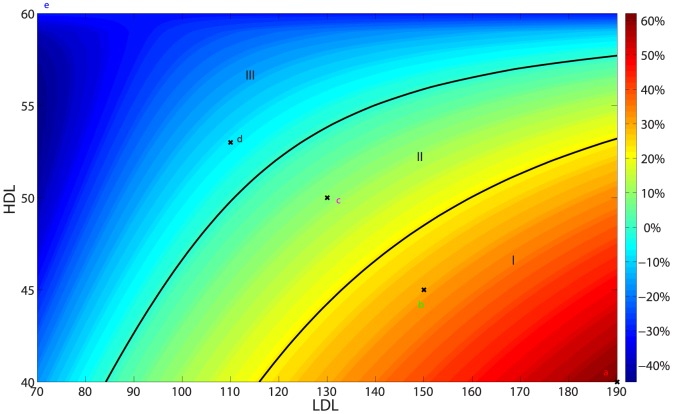
Risk map for plaque development: Region I high risk; Region II low risk; Region III no risk. The five points 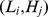 whose plaque's weight was simulated in Fig. 7 over a period of 300 days are indicated by “x”.

Fig. 8 shows a risk-map of plaque development. To create the risk-map, we divided the LDL axis by 121 equidistant points, i.e, 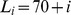 (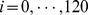), and divided the HDL axis by 21 equidistant points, i.e., 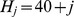 (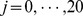). For each pair 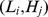, we computed the weight of the plaque, 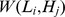 after 100 days on the domain shown in Fig. 3 (B), and formed the risk matrix
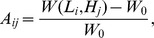
where 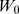 is the initial weight of the plaque. The vertical axis on the right of Fig. 8 shows the legend of the percentage of plaque growth or shrinkage. Accordingly, we divided the LDL-HDL plane into three regions: region I predicts high risk of plaque development, region III predicts no risk, and the intermediate region II predicts low risk.

### Sensitivity analysis

In order to support the robustness of the simulation results, we ran sensitivity analysis on parameters which appear in the differential equations and in the boundary conditions. The parameters chosen are those whose baseline was somewhat crudely estimated while at the same time they seem to play an important role in the development of the plaque. Specifically, we chose all the 15 production rate parameters from the third box of Table 4, all the 5 influx rate parameters from the third box of Table 5, and 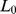, 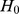. We list all these parameters with their range, baseline and unit in Table 6.

**Table 6:** Parameters chosen for sensitivity analysis.

Parameter	Range	Baseline	Unit
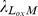	[5,20]	10	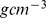 day^−1^
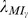	[0.002, 0.01]	0.005	day^−1^
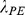	[ 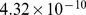 , 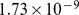 ]	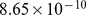	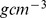 day^−1^
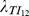	[ 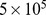 , 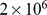 ]	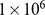	day^−1^
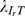	[0.033, 0.132]	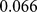	day^−1^
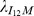	[ 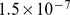 , 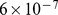 ]	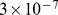	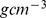 day^−1^
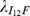	[ 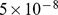 , 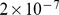 ]	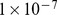	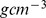 day^−1^
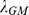	[0.05, 0.2]	0.1	day^−1^
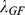	[0.016, 0.066]	0.033	day^−1^
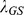	[0.25, 1]	0.5	day^−1^
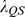	[ 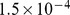 , 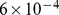 ]	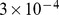	day^−1^
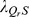	[ 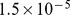 , 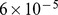 ]	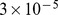	day^−1^
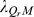	[ 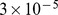 , 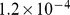 ]	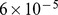	day^−1^
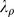	[0.266, 0.864]	0.432	day^−1^
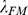	[0.06, 0.24]	0.12	day^−1^
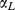	[0.5, 2.0]	1.0	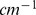
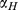	[0.5, 2.0]	1.0	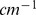
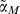	[0.1, 0.4]	0.2	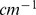
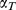	[0.025, 0.1]	0.05	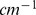
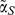	[0.1, 0.4]	0.2	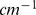
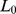	[ 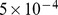 , 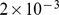 ]	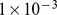	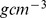
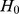	[ 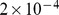 , 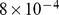 ]	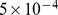	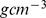

**Figure 9:**
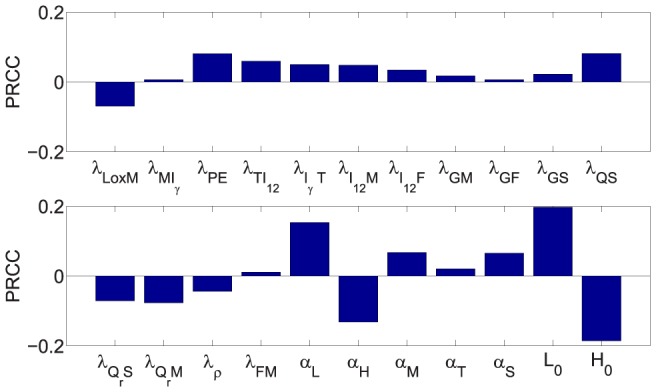
The PRCC of parameters for sensitivity analysis.

Following the sensitivity analysis method described in [53], we performed Latin hypercube sampling and generated 100 samples to calculate the partial rank correlation coefficients (PRCC) and p-values with respect to the weight of the plaque after 300 days. The PRCCs are shown in Fig. 9, and all the p-values (not shown here) are less than 0.01. A positive PRCC (i.e., positive correlation) means that an increase in the parameter value will increase the weight of the plaque while a negative PRCC (i.e., negative correlation) means increase in the parameter will decrease the weight of the plaque. We note that 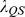 is positively correlated, as it should be. Indeed, if 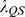 is increased then MMP (*Q*) is increased (Eq. (12)) so that ECM (*ρ*) is decreased (Eq. (19)) and hence the plaque weight 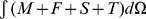 is increased (Eq. (16)). As another example, note that 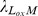 is negatively correlated. Indeed, if 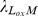 is increased then 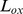 is decreased (Eq. (3)), and 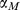 in the boundary condition will decrease, leading to smaller *M*, and then to smaller *T* and *F*. Similar explanation can be given to the other parameters.

The most significant positively correlated parameters are 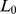 and its influx rate 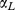. This is not surprising since LDL initiates the plaque formation. The most significant negatively correlated parameters are 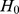 and its influx rate 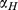. Indeed, since HDL reduces the availability of free radicals, it plays an important negative role in plaque formation.

## Discussion

Atherosclerosis is a disease in which a plaque builds up inside an artery. The process of plaque formation begins when, as a result of a lesion in the artery, cholesterols LDL and HDL enter the intima, and LDL becomes oxidized by free radicals. Upon sensing ox-LDL, endothelial cells secrete MCP-1 which attracts monocytes from the blood. As monocytes enter to the intima, they differentiate into macrophages that ingest the ox-LDL and become foam cells. Foam cells attract more macrophages, followed by T cells from the blood, and SMCs from the media. HDL reduces the available free radicals, as well as inflammation within the evolving plaque, thus HDL acts to block plaque growth.

Public health guidelines in the U.S. specify that LDL level of 100–129 mg/dL is near ideal, 130–159 mg/dL is borderline high, and 160–189 mg/dL is very high, whereas concentration of HDL above 60 mg/dL is best, and below 40 mg/dL for men or below 50 mg/dL for women is poor [25]. An important question is how to evaluate the risk of atherosclerosis for a pair of LDL and HDL taken together. This question is addressed in the present paper. We built a mathematical model of plaque development by a system of partial differential equations. The model includes two parameters: 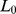, the level of LDL in the blood, and 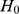, the the level of HDL in the blood.

The model can simulate the evolution of a small plaque for any pair of values of 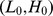. In Figs. 4, 5, 6, we simulated the plaque evolution over a period of 300 days. For example, one extreme case of 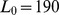 mg/dL, 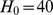 mg/dL, the plaque doubled after 300 days; in another extreme case of 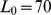 mg/dL, 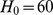 mg/dL, the plaque disappeared after 300 days. We created a risk-map by taking sampling points of LDL and HDL values, and computing the weight of the plaque for each pair 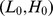 after 100 days. The map shown in Fig. 8, indicates the percentage of plaque growth or shrinkage for any such pair. We accordingly divided the (LDL,HDL) quadrant into three regions: high risk, low risk, and non risk.

The need to consider the ratio of LDL/HDL in predicting coronary heart disease was suggested in a case study by [54]. The American Heart Association considers the ratio 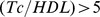 to indicate high risk of heart disease, and the ratio 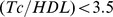 to be risk free [55], where Tc denotes the total cholesterol, which is calculated by the formula
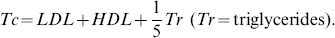

Table 7 shows the National Cholesterol Education Program (NCEP) guidelines associated with plaque buildup [56]. Accordingly, for the five points (a)–(e) in **Results** we have:

**Table 7:** National Cholesterol Education Program guidelines.

LDL Cholesterol Level	Category
Less than 100 mg/dL	Optimal
100 to 129 mg/dL	Near or above optimal
130 to 159 mg/dL	Borderline high
160 to 189 mg/dL	High
190 mg/dL and above	Very high

HDL Cholesterol Level	Category
Less than 40 mg/dL (for men)	Low HDL cholesterol.
Less than 50 mg/dL (for women)	A major risk factor for heart disease.
60 mg/dL and above	High HDL cholesterol.

Triglyceride Level	Category
Less than 100 mg/dL	Optimal
Less than 150 mg/dL	Normal
150–199 mg/dL	Borderline high
200–499 mg/dL	High
500 mg/dL and above	Very high

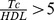
 for any value of Tr;

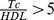
 if Tr is above normal;

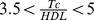
 if Tr is not very high;

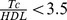
 if Tr is normal;

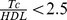
 if Tr is normal;.

According to the NCEP guidelines, (a) and (b) should be in the high risk region; (c) in the low risk region; and (d), (e) in the no risk region, as indeed they are according placed in the risk map in Fig. 8.

Some anti-cholesterol drugs, such as statins, lower LDL and at the same time also increase the HDL [24]. It is important to know which drugs can best achieve the desired risk-free balance between LDL and HDL, that is, bring the individual's (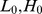) into the no risk (or low risk) region. By focusing not on just reducing 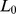 or on just increasing 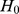, but on moving the combined (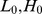) to the no risk (or low risk) region in the shortest medically feasible path, we believe one could choose a more personalized medicine from those currently available, which will reduce the risk of atherosclerosis with the lowest amount of doze, thereby also possibly reducing potential negative side effects.

**Figure 10:**
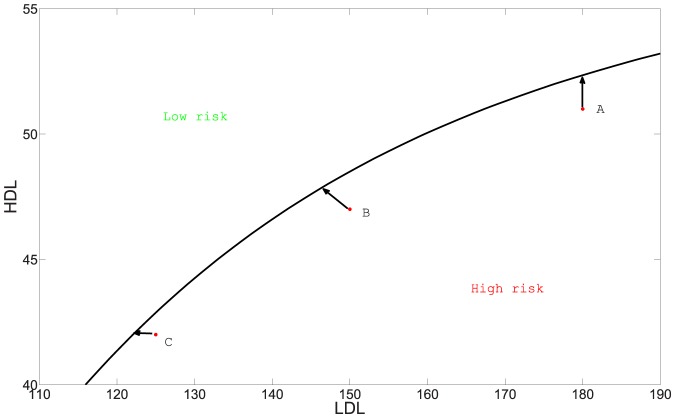
Drug treatment recommended for individuals A, B and C.

To illustrate this approach, we note that for some drugs the ratio of decrease in LDL to increase in HDL is already known. For example, this ratio is 1/3 for the new experimental drug *Evacetrapib*. Some anti-cholesterol drugs only decrease LDL (e.g. Colestid) while others only increase HDL (e.g. Lofibra). We can the represent effect of such drugs by unit vectors in the 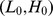 plane: for example, Colestid ←, Lofibra ↑, and Evacetrapid 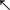. In Fig. 10. we consider three individuals, A, B, and C in the high risk region. In order to move them to the low risk region with the minimum amount of medication (side effects are ignored), the individual should choose the drug for which the line segment from the individual initial position to the low risk region is the shortest (We assume that the amount of drug is proportional to the length of the line segment). Thus the best drug for A is the one that primarily increases HDL. Similarly, C will do better with a drug that primarily decrease LDL, and B should use a drug with appropriate ratio of decreasing LDL to increasing HDL.

Some work has been done on antioxidant therapy for reducing the risk of atherosclerosis, but so far it has had limited success in preventing cardiovascular diseases [57]–[59]. A review of studies in which antioxidant gene therapy has been successfully used is given in [60]. Our model could account for antioxidative medication once we gain a good understanding of how such medication affects the source of free radicals, *r*_0_, in Eq. (4).

Some of the parameters in the differential equations in our model had to be rather crudely estimated, since no data were available, while others may slightly vary depending on the individual. As more data become available, parameter values may be further refined. Our model uses only the values of LDL and HDL as biomarkers. It will be interesting in the future to incorporate also triglycerides into the risk-map. Future work should also explore how other risk factors, such as high blood pressure, smoking and diabetes affect the risk-map.

We did not include in this paper the circulation ox-LDL in the blood, which is elevated only in patients with advanced atherosclerosis [61], [62]. Our model could be extended to include this additional biomarker, but at present there is not enough data on how the level of ox-LDL in the blood correlates to a specific advanced state of the disease.

## Supporting Information

Algorithm S1 Algorithm for finite element implementation of the mathematical model.
(PDF)

Figure S1 Macrophages population for different levels of LDL and HDL.
(PDF)

Figure S2 SMCs population for different levels of LDL and HDL.
(PDF)

Figure S3 T cells population for different levels of LDL and HDL.
(PDF)

Figure S4 Foam cells population for different levels of LDL and HDL.
(PDF)

Figure S5 Concentration of ox-LDL for different levels of LDL and HDL.
(PDF)

Figure S6 Concentration of IFN-*γ* for different levels of LDL and HDL.
(PDF)

Figure S7 Concentration of IL-12 for different levels of LDL and HDL.
(PDF)
