# Supplementary material for: The LDL-HDL Profile Determines the Risk of Atherosclerosis: A Mathematical Model
Source: PLoS One. 2014 Mar 12;9(3):e90497. doi: 10.1371/journal.pone.0090497 (PMC3951264; doi:10.1371/journal.pone.0090497)
Supplement: Algorithm S1 — Algorithm for finite element implementation of the mathematical model. (PDF) [file pone.0090497.s001.pdf]

Algorithm S 1: Algorithm for implementation

Input : The initial condition, time stepsize τ, the maximum time-steps N_T, step size for remeshing M.

Generate a triangular mesh over the domain.

for $n = 0 : N_T$ **do**

- Compute L^{n+1}, H^{n+1}, r^{n+1}, L_{ox}^{n+1}, M^{n+1}, P^{n+1}, T^{n+1}, I_{γ}^{n+1}, S^{n+1}, I_{12}^{n+1}, G^{n+1}, Q^{n+1}, Q_r^{n+1}, ρ^{n+1}, F^{n+1} by solving equation (33);
- Compute the mean curvature of Γ_I^n, κ^n;
- Compute σ^{n+1} by solving equation (34);
- Compute V_n in equation (25) and update Γ_I^{n+1};
- Remeshing every M steps.

end
