# Supplementary figures and images for: The LDL-HDL Profile Determines the Risk of Atherosclerosis: A Mathematical Model
Source: PLoS One. 2014 Mar 12;9(3):e90497. doi: 10.1371/journal.pone.0090497 (PMC3951264; doi:10.1371/journal.pone.0090497)

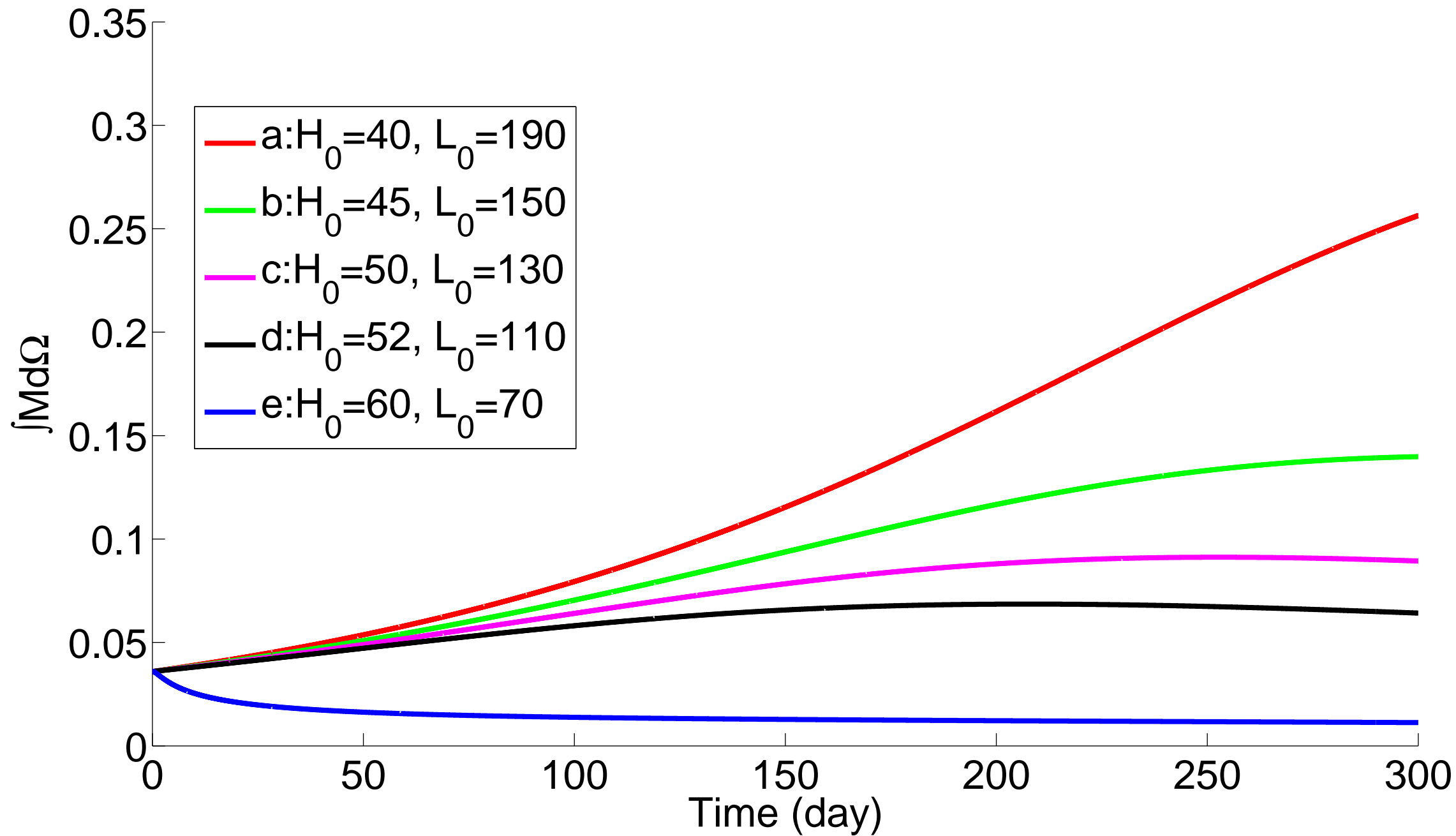

Supplement: Figure S1 — Macrophages population for different levels of LDL and HDL. (PDF) [file pone.0090497.s002.pdf]

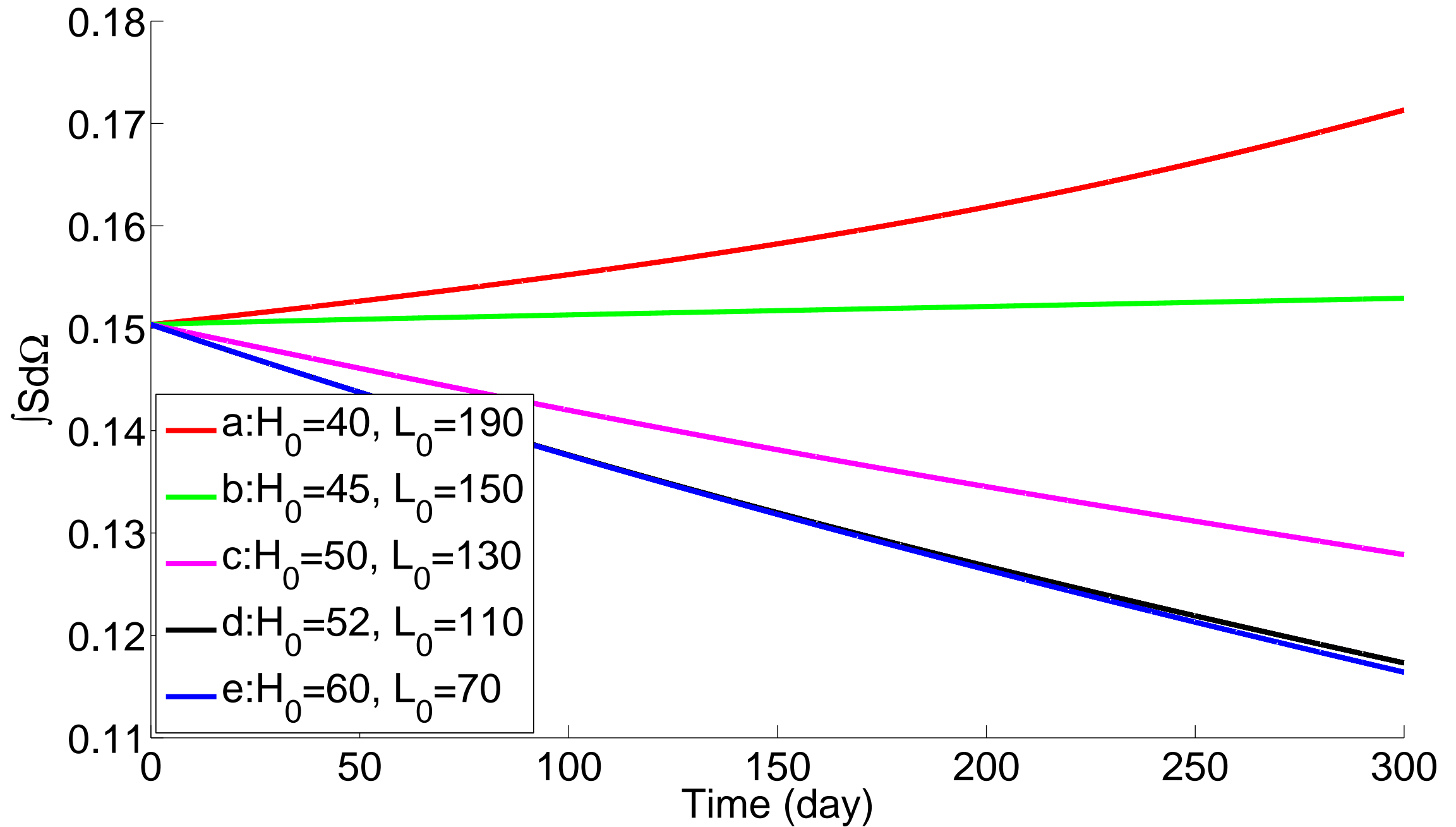

Supplement: Figure S2 — SMCs population for different levels of LDL and HDL. (PDF) [file pone.0090497.s003.pdf]

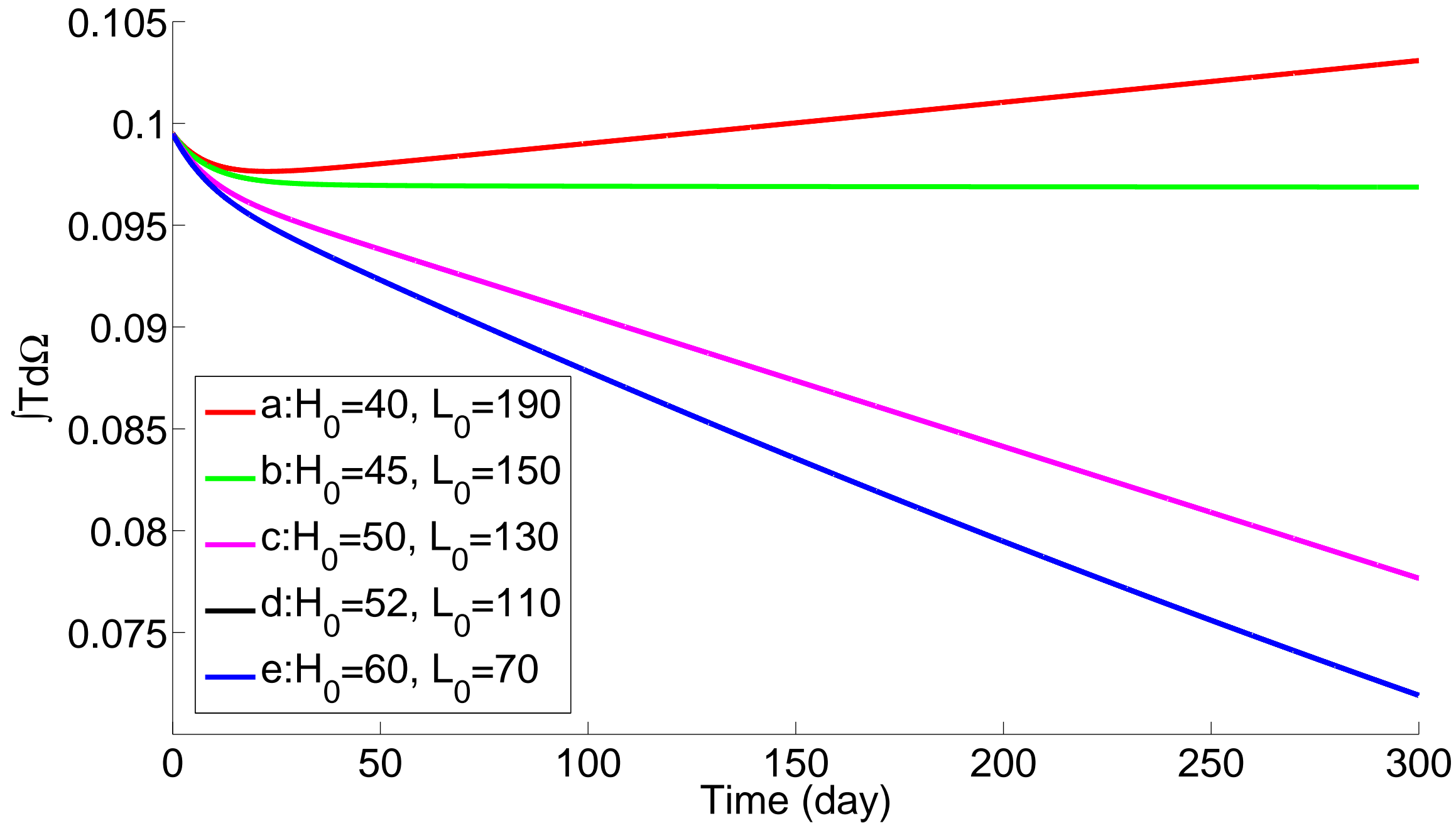

Supplement: Figure S3 — T cells population for different levels of LDL and HDL. (PDF) [file pone.0090497.s004.pdf]

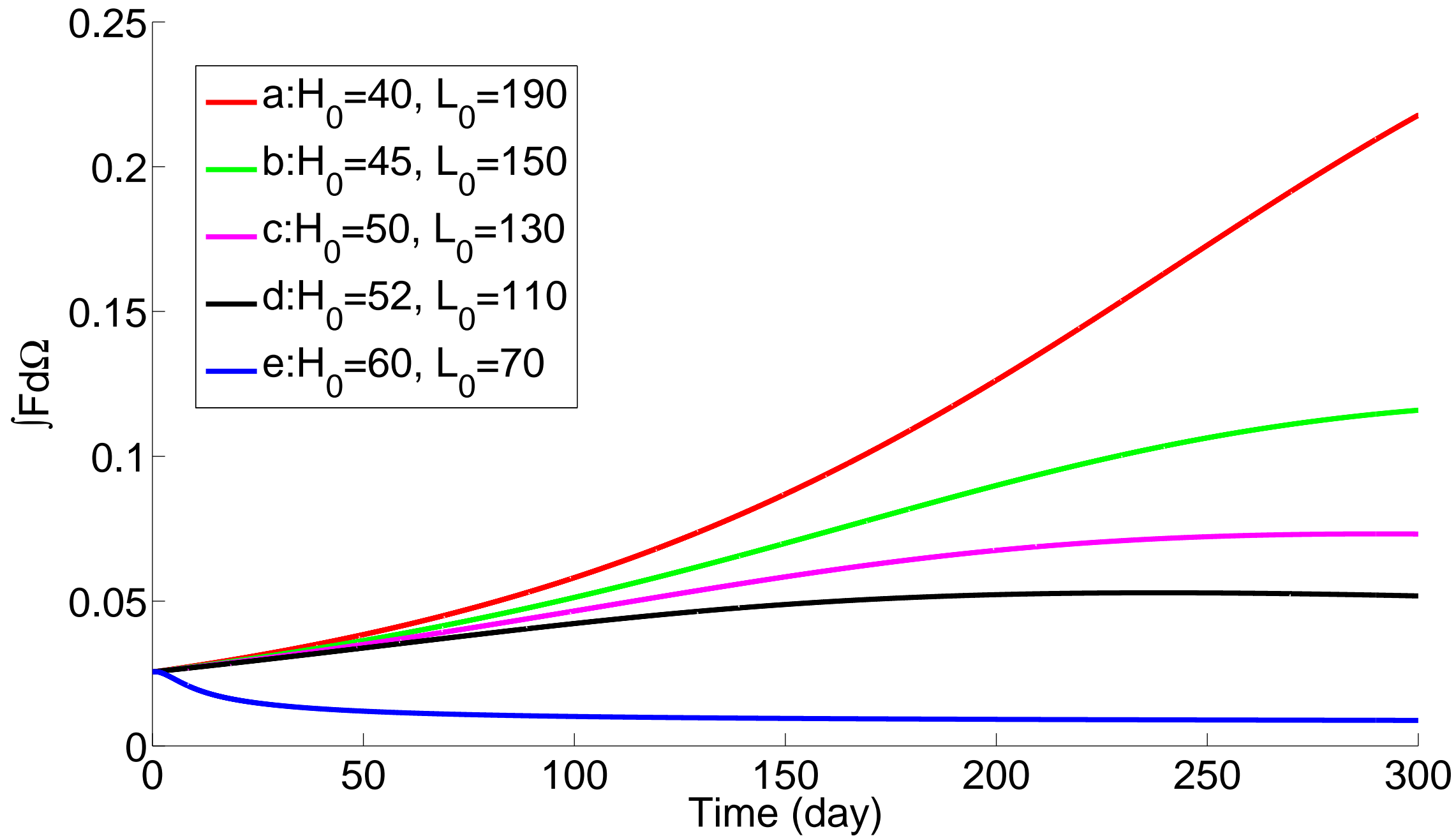

Supplement: Figure S4 — Foam cells population for different levels of LDL and HDL. (PDF) [file pone.0090497.s005.pdf]

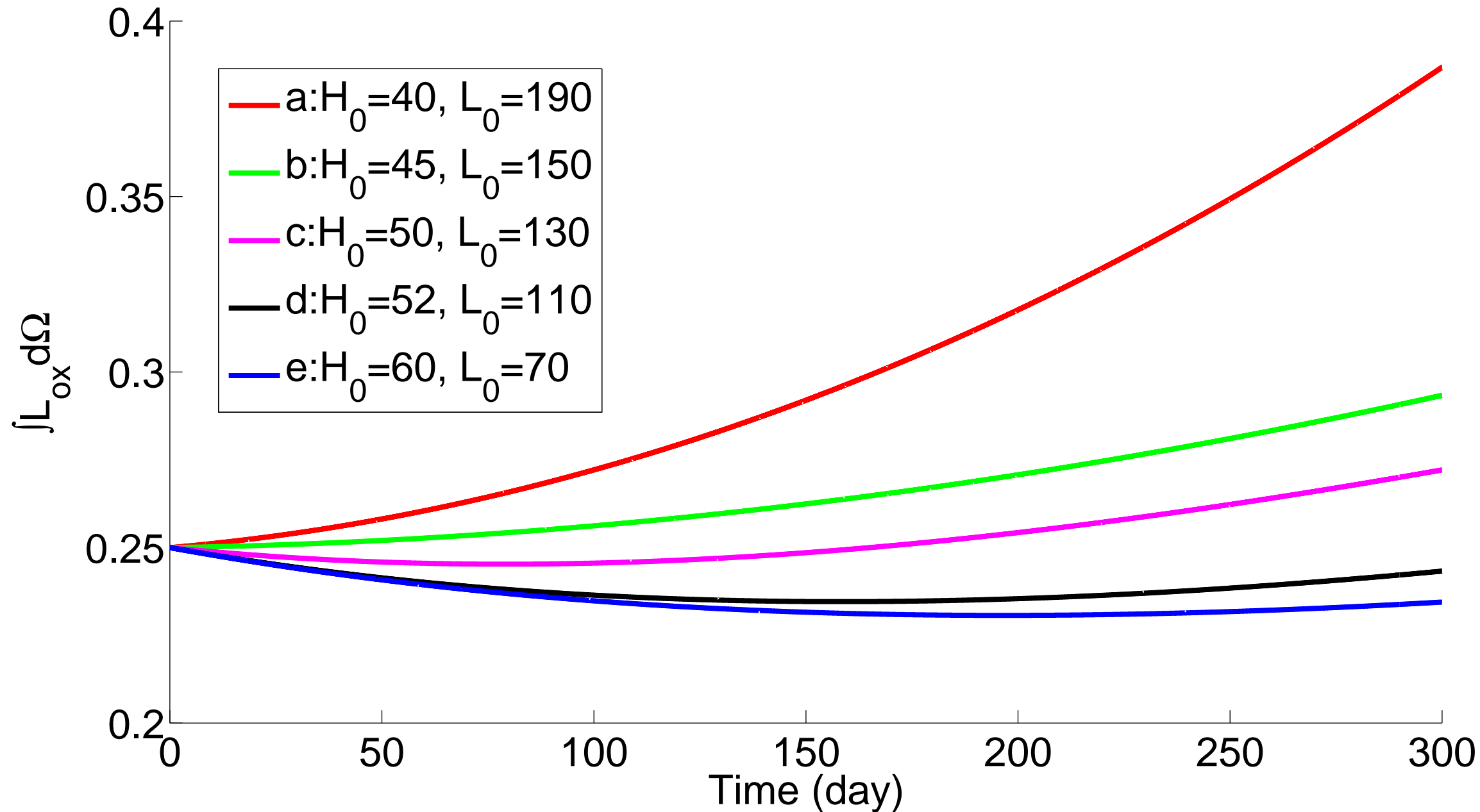

Supplement: Figure S5 — Concentration of ox-LDL for different levels of LDL and HDL. (PDF) [file pone.0090497.s006.pdf]

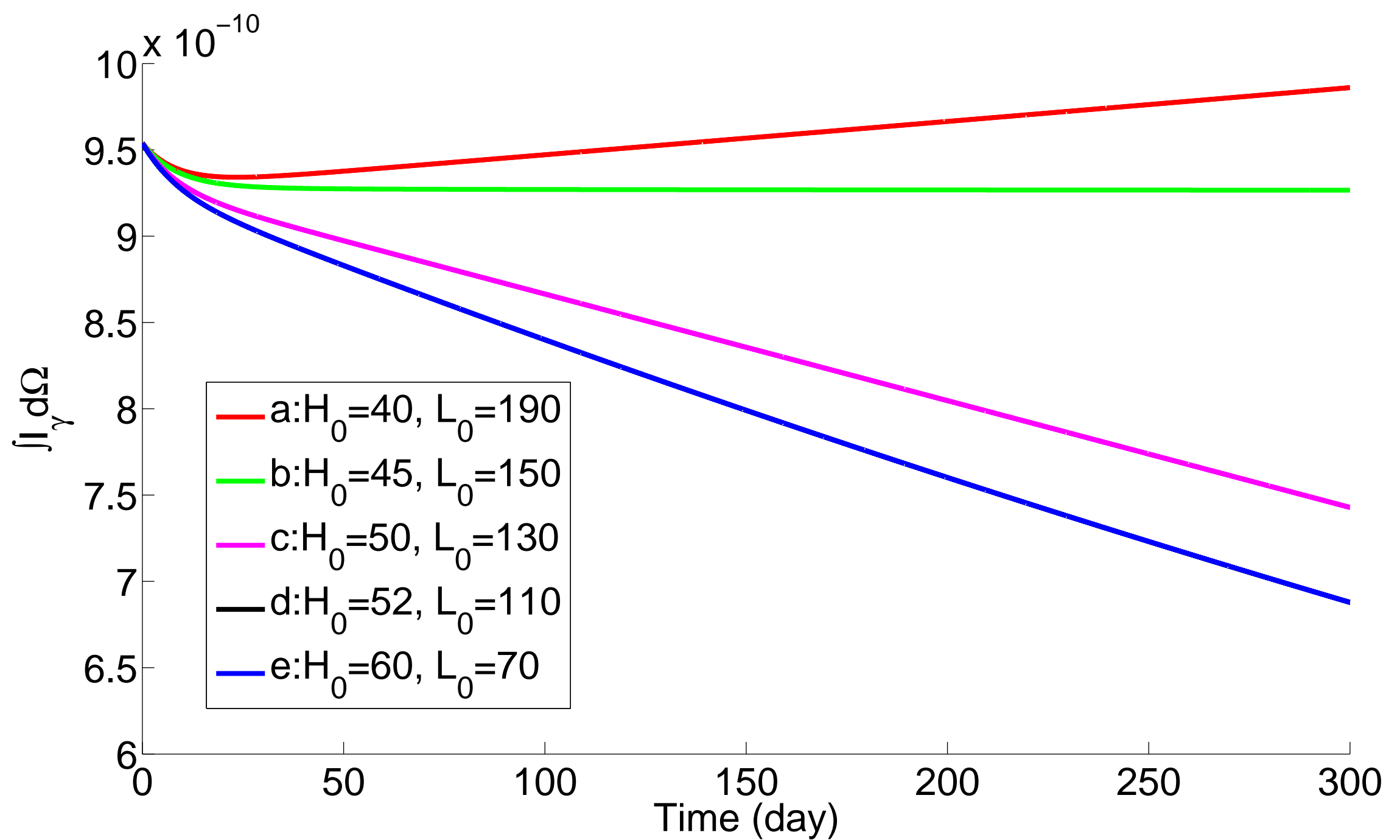

Supplement: Figure S6 — Concentration of IFN-γ for different levels of LDL and HDL. (PDF) [file pone.0090497.s007.pdf]

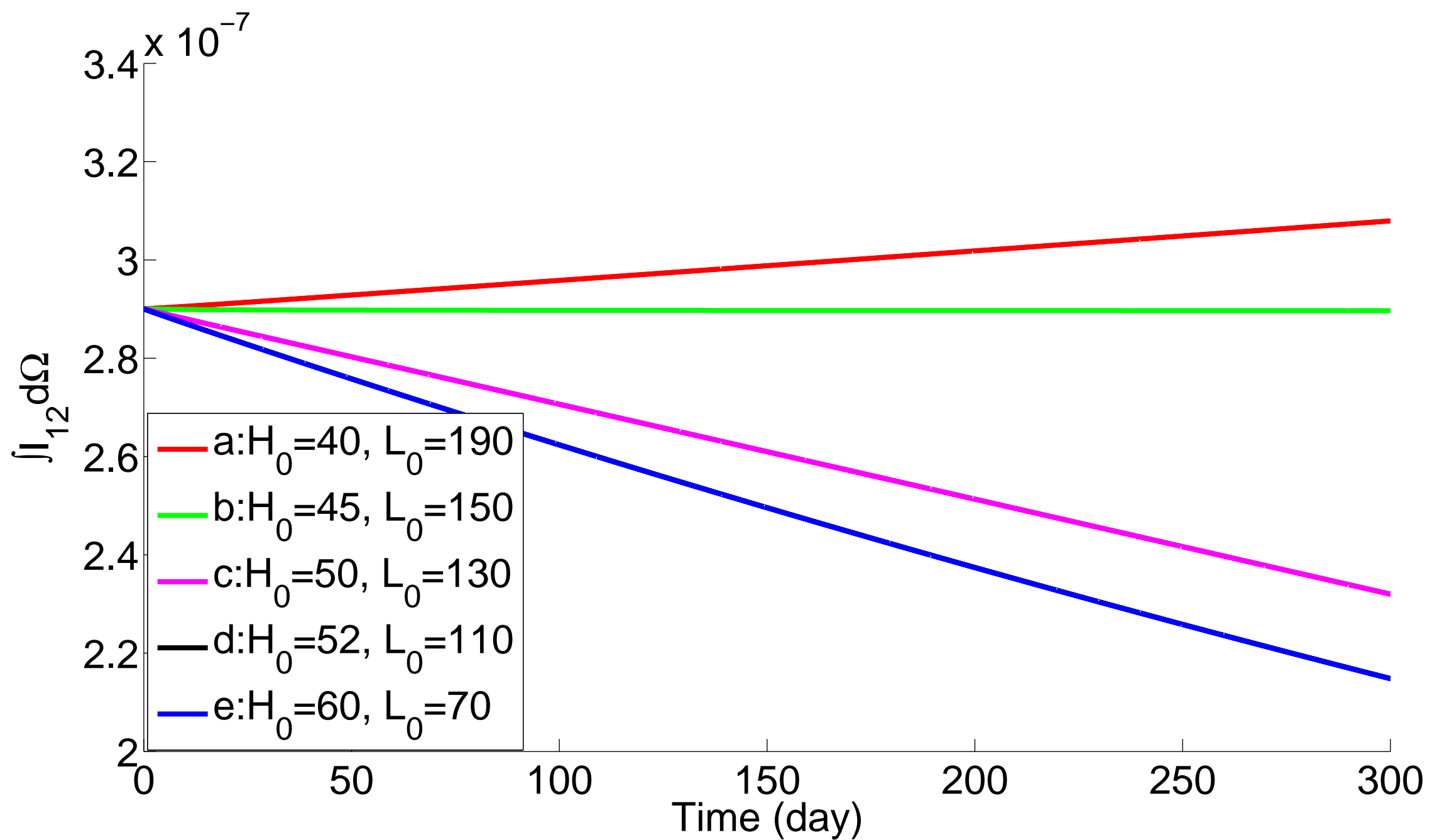

Supplement: Figure S7 — Concentration of IL-12 for different levels of LDL and HDL. (PDF) [file pone.0090497.s008.pdf]
